# Hereditary predisposition to malignant myeloid hemopathies: Caution in use of saliva and guideline based on our experience

**DOI:** 10.3389/fonc.2023.1120829

**Published:** 2023-02-27

**Authors:** Alexandre Perani, Sylvie Bourthoumieu, David Rizzo, Jasmine Chauzeix, Benjamin Dauriat, Pascal Turlure, Stéphane Girault, Léa Veyrune, Maxime Roubinet, Jean Feuillard, Catherine Yardin, Nathalie Gachard

**Affiliations:** ^1^ Laboratoire de Cytogénétique et Génétique Médicale, Centre Hospitalier Universitaire CHU de Limoges, Limoges, France; ^2^ Laboratoire d’Hématologie, Centre Hospitalier Universitaire CHU de Limoges, Limoges, France; ^3^ Service d’Hématologie Clinique, Centre Hospitalier Universitaire CHU de Limoges, Limoges, France

**Keywords:** myeloid hemopathies, predisposition, saliva, *DDX41*, genetic counseling

## Abstract

**Background:**

Predisposition to myeloid malignancies is a field at the border of hematology and genetics. Knowledge in this domain has so rapidly increased that WHO defined in 2016 the new “Myeloid Neoplasms with Germline Predisposition” category of tumors. High throughput sequencing is frequently performed in tumors either for diagnosis or prognosis, but this approach may identify potential germline variants that have to be confirmed on non-infiltrated tissues.

**Method:**

In this study, we systematically compared NGS data from genetic analysis performed on all sample types (bone marrow, blood, saliva, skin fibroblasts and hair follicles) in 29 patients, and 44 of their relatives (blood and saliva).

**Results:**

We showed that saliva was usable for relatives, but only for 24% (7/29) of our patients. Most of patients’ saliva were either “non-contributive” (14/29 *i.e.*, 48% because clearly or probably infiltrated) or “inconclusive” (8/29 corresponding to 28%).

**Conclusion:**

The recommendations for the use of saliva we present here focus on the importance of collecting saliva during remission when possible. Moreover, we propose hair follicles as an alternative to skin biopsy, that remains the gold standard especially in case of allogenic hematopoietic stem cells transplantation. Technological progresses have revolutionized the diagnosis of predisposition to solid or hematological malignancies, and it is very likely that new techniques will help to manage the familial predisposition in the future.

## 1 Introduction

Hematopoiesis is the biological lifelong process that produces all blood cells from a very small contingent of multipotent stem cells located in the bone marrow. That differentiation is closely regulated by numerous factors (1). Germline (GL) mutations in genes involving this pathway increase the risk of developing hemopathies, leading World Health Organization (2) and International Consensus Classification (3) to define the myeloid neoplasms (MN) “with germline predisposition” subtype.

Among these genes, recent literature shows an association between germline *DEAD-Box Helicase 41 (DDX41)* mutations and familial MN, including myelodysplastic syndromes (MDS) (4, 5). Recent study of Makishima et al. (6) and the review of Kim et al. (7) characterized *DDX41-*mutated MN, detailing their high frequency among MN with germline predisposition (about 80%), specific clinical outcomes (high progression rate to acute myeloid leukemia: AML) and high penetrance of GL traits of *DDX41* (about 50% by the age of 90), that even tend to consider them as a specific subset of MN.

MDS is an heterogenous group of clonal myeloid disorders. They are usually suspected in presence of unexplained persistent peripheral blood cytopenias, but diagnosis requires a bone marrow assessment. The presence of dysplasias on cellular morphological examination is a hallmark of the diagnosis (8).

Three groups of factors can help to define the prognosis of MDS: cytogenetics aberrations, bone marrow blast percentage and depth of cytopenia. They are combined in the Revised International Prognosis Scoring System (IPSS-R) widely used in clinical routine. It assesses the risk of secondary evolution AML (9). Nevertheless, studies of genomic alterations in tumors become important in the diagnostic process, not only in solid tumors but also in hematological malignancies (HM). Based on National Comprehensive Cancer Network (NCCN) constatations (10), Bernard et al. (11) recently proposed an adaptation of IPPS-R, called IPSS-M score (M for molecular), including molecular abnormalities of 31 genes.

In this context, High Throughput Sequencing (HTS) of gene panel involved in HM is frequently performed in early stage of MDS. In some cases, this approach on bone marrow sample may identify potentially germline variants. Confirmation of their germline nature on another biological tissue becomes therefore essential for appropriate clinical management and genetic counseling for relatives.

Due to diffusion of hematopoietic tumoral cells in peripheral blood, saliva appears to be a more appropriate biological sample for germline studies, and its collection is not invasive. Nevertheless, literature reports risks of saliva’s contamination by tumoral blood cells (12, 13); and eventuality that could impede the discrimination between germinal and acquired mutations (14). To our knowledge, no recent study is available to drive geneticists’ routine practice.

The aim of this study is to report our local experience and propose recommendations for use of saliva in case of suspicion of familial hemopathy (FH). Here, we compared HTS data from saliva, bone marrow, and other tissues (blood, cultured fibroblasts from skin biopsy, or hair follicle) collected for diagnosis of FH or follow-up. Data from patients as well as those collected in their relatives were also used to build the guideline we propose.

## 2 Material and method

### 2.1 Patients and relatives’ inclusion

We extracted from our database all analysis performed between 2020 and 2022 on DNA isolated from saliva (n=78). Patients were distinguished from relatives through a systematic review of medical history and molecular analysis performed in our laboratory (**Supplementary Figure S1**). We excluded 5 patients because HTS data at diagnosis were performed elsewhere and/or unavailable. Finally, 29 index-cases and 44 relatives were included. Absence of hemopathy in relatives was verified before sample collection, with standard blood count and clinical examination.

All individuals received a genetic counselling from a geneticist of Limoges University Hospital Center. They all provided informed consent for molecular studies, and study was approved by local ethic committee (registration number: 584-2022-240).

### 2.2 Molecular studies

We isolated DNA with Maxwell RSC instrument (Promega, Madison, WI, USA) using the appropriate kit for each sample type according to the supplier’s recommendations. DNA was extracted from saliva (collected with ORAGEN DNA Kit, DNA Genotek, Kanata, Canada) with the Stabilized Saliva DNA kit (Promega); from whole blood on EDTA or 5M cell pellets with the Simply DNA Blood kit (Promega); and from hair follicle (10 follicles minimum) with the Tissue and Hair kit (Promega). We only extracted DNA manually from fibroblasts with the PureGene kit (QIAGEN, Hilden, Germany), according to manufacturer’s recommendations. We cultured fibroblasts after microdissection of skin biopsy in incubator (37°C, 5% CO2), with Chang Medium (Clinisciences, Nanterre, France).

We prepared amplicon library using an Ion AmpliSeq custom panel (ThermoFisher, Waltham, MA, USA) targeting 17 diagnostic and prognostic genes (list in **Table S1**). HTS was then performed using the Ion S5 Sequencing System (ThermoFisher). Alignment and variant calling were made with Torrent Suite Software (ThermoFisher), and annotation with VEP and Ensembl. Copy Number Variations (CNV) were detected with CovCopCan (15) and OncoCNV (16). We classified SNV according ACMG recommendations. Sensitivity of SNV with this method is greater than 99% for minimal depth of 100X and analysis detects CNV if present in greater than 30% of total sample cells.

Sanger method was performed to sequence gene regions with depth <100X as well as mutation specific analysis in patents and their relatives. We performed amplification with Taq Purple Mix (Ozyme, ST CYR L’ECOLE, France), purification of PCR products with ExoSap (Applied biosystems, Waltham, MA, USA) and sequencing with BigDye Terminator V1.1 (Applied Biosystems) on 3130xL or 3500 xL Genetic Analyzer (Applied Biosystems).

### 2.3 Contribution of saliva collected

We systematically reviewed results of HTS analysis in all samples (bone marrow at diagnosis and for follow-up, blood, skin fibroblasts, hair follicle and saliva) for each patient. Variant Allele Frequencies (VAF) of pathogenic (class 5) and likely pathogenic (class 4) variants was compared between samples. We considered that saliva was « not contributive » for germline studies when somatic mutations present at diagnosis were also detected in saliva regardless of their VAF, suggesting an infiltration by tumoral cells. Conversely not infiltrated saliva were qualified as “contributive”, and if it was not possible to affirm the absence of infiltration saliva were “inconclusive”. Moreover, for patients treated with allogenic hematopoietic stem cell (HSC) transplantation, we studied the presence or absence and zygosity of Single Nucleotide Polymorphism (SNP) to assess donor’s contribution to HTS results in patients.

## 3 Results

### 3.1 Patients and mutations

Between May 2020 and May 2022, 29 patients and 44 relatives (of 14 patients) were included in the study. Majority of them were males (respectively 21 in the group “patient”, and 20 in the group “relatives”), but ages were similar (59 years ± 14 *versus* 53 years ± 15 respectively). HTS was performed for diagnosis and prognosis in patients with various types of myeloid disorders (**Table 1**). Myelodysplastic syndromes (all types) were the most frequent (18/29, 62%).

**TABLE 1:** Myeloid disorders of patients included in the study.

Myeloid Disorder			Sex		Age mean (± SD)
			Male	Female	
**MDS**	MDS *ns*	3	2	1	63 (50-75)
	MDS-EB *ns*	1	0	1	77
	MDS-EB1	8	7	1	64 (55-73)
	MDS-EB2	5	4	1	62 (56-67)
	RCMD	1	1	0	57
	*Total*	18	14	4	63 (55-71)
**AML**	AML *ns*	6	4	2	60 (45-75)
	AML1	1	1	0	46
	AML2	2	1	1	38 (35-41)
	*Total*	9	6	3	59 (44-74)
**Other**	CMML	2	1	1	59 (44-75)
***Total***		29	21	8	59 (45-73)

MDS, Myelodysplastic Syndrome; ns, not specified; EB, Excess Blasts; RCMD, Refractory Cytopenia with Multilineage Dysplasia; AML, Acute Myeloid Leukemia; CMML, Chronic Myelomonocytic Leukemia.

Patients received genetic counselling for suspicion of germline mutation of 6 genes: *CBL* (n=1)*, DDX41* (n=22)*, GATA2* (n=1)*, KRAS* (n=1)*, RUNX1* (n=2), and *TP53* (n=2). Among them, *DDX41* was thus the most frequently mutated, but only five pathogenic mutations were detected: G173R (n=12), A270V (n=1), E268Dfs*36 (n=3), L283Cfs*21 (n=4) and S363del (n=2). All of them were already reported (6, 17, 18).

### 3.2 Saliva contribution

Clinical data and sequencing results of patients and their relatives are presented in **Table S2**. Results obtained with Sanger sequencing on saliva and blood in relatives were concordant. We thus concluded that saliva was contributive and usable in clinical routine for them, after verification of absence of hemopathy.

#### 3.2.1 Contributive saliva samples

In the group of 29 patients, saliva samples were contributive in 7 patients, corresponding to 24% of our cohort (patients ID 2, 9, 11, 14, 24, 28 and 29). All samples were collected at remission (after allogenic HSC transplantation or chemotherapy) or during treatment, and HTS did not detect somatic mutation in saliva. Among them, only patient ID 11 beneficiated of Sanger sequencing of *DDX41* to search for S363del mutation, although somatic mutations were present at diagnosis (*IDH2* R140Q and *DNMT3A* R882H). We however considered that saliva as “contributive”, because *IDH2* and *DNMT3A* mutations were absent of NGS performed on bone marrow for follow-up one month later, suggesting that saliva was not infiltrated when it was collected.

#### 3.2.2 Non-contributive saliva samples

Ten samples (34%) were clearly (patients ID 1, 6, 10, 13, 16, 25 and 27) or probably (ID 3, 8 and 23) infiltrated by tumoral cells. Infiltration was demonstrated by presence of somatic mutations detected at diagnosis in saliva or in samples collected for follow-up (blood or bone marrow). Concerning patient ID 8 and 23, saliva infiltration was highly probable, because collected during a progressing disease (ID 8) or before a transplantation of HSC and out of remission (ID23). Among them, three patients (ID 13, 23 and 27) were treated with allogenic HSC transplantation, but their saliva samples were collected before, which did not bias our interpretations (no contribution of donor to results in these patients).

Surprisingly, four other saliva samples were also not-contributive even though collected after allogenic HSC transplantation (patients ID 4, 12, 21 and 22). Thus, the total of not-contributive saliva was 48% (14 among 29). Conclusions for those patients required more investigations so we reviewed presence or absence of SNP detected by NGS in the different samples available.

**Figure 1:**
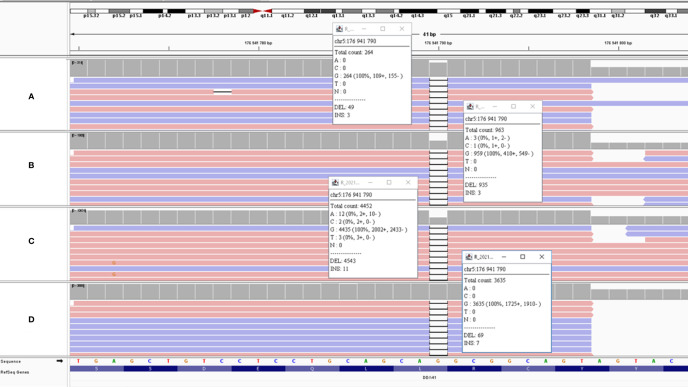
IGV view of DDX41 E268Dfs*21 mutation in different sample type of patient 4. **(A)** Saliva (VAF = 15.8%); **(B)** Skin fibroblasts (VAF=50%); **(C)** Bone marrow at diagnosis (VAF= 51.2%); **(D)** = bone marrow at relapse (VAF=1.9%). VAF of the mutation on saliva showed a chimerism, and germline nature is proven by analysis of skin fibroblasts.

- Patient 4: two *DDX41* mutations were present at diagnosis: E268Dfs*21 (VAF=51,2%) and G530D (VAF=3,5%). Bone marrow collected for relapse despite an extra-familial allogenic HSC transplantation, found same mutations (VAF= 1.9% and 1.6% respectively) and the new *IDH1* R132C mutation (VAF= 3.5%). In saliva collected 5 months later, we detected *DDX41* E268Dfs*28 mutation, with a VAF of 15.8% consistent with an incomplete chimerism (**Figure 1**). We confirmed that hypothesis with comparison of SNP which showed that 9 SNP detected in saliva and bone marrow at relapse were absent of bone marrow collected at diagnosis (**Table S3**). Finally, we could affirm the germline nature of the *DDX41* E268Dfs*28 mutation with NGS performed on DNA isolated from skin fibroblasts (VAF=50%, and absence of the 9 SNP from the donor).
- Patient 12: difficulties of interpretation were comparable to situation of case 4, but this patient received two allogenic HSC transplantations: the first with a relative and the second with an unrelated donor. The *DDX41* G173R detected at diagnosis and relapse after the first transplantation was not detected on blood and saliva after the second graft. HTS performed on skin fibroblasts and hair follicle confirmed the germline nature of *DDX41* G173R mutation (VAF=54% and 48.8% respectively). Comparisons of SNP with skin (data on request) showed that i) the relapse was developed on transplant (different SNP profile between skin and bone marrow at relapse); and ii) only DNA of unrelated donor is present in saliva, corresponding to a 100% donor chimerism (different SNP profile between skin and saliva).
- Patients 21 and 22: Situation for these patients was quite different of previous patients. *DDX41* G173R mutation was still present after one (patient 21) or two (patient 22) transplantations, but with a very low VAF in saliva (4% and 5% respectively), suggesting an incomplete chimerism. We confirmed this hypothesis with SNP studies (**Table S3**) which showed presence of some patient’s SNPs (detected in skin) in saliva with a low VAF. NGS performed on skin fibroblasts confirmed germline nature of *DDX41* G173R mutation in these patients.

#### 3.2.3 Inconclusive saliva samples

The eight remaining samples (28%) were considered as “inconclusive” because it was not possible to conclude with the available data (patients ID 5, 7, 15, 17, 18, 19, 20 and 26). None of these saliva samples were collected at remission, and bone marrow at diagnosis showed presence of clonal and subclonal mutations. Our local workflow initially considers only Sanger sequencing on saliva to detect supposed germline mutations. Majority of these eight cases (ID15, 17, 19, 20 and 23) were characterized by a *DDX41* acquired mutation in minor proportion of cells in bone marrow (VAF ranged from 3 to 16%), thus probably not detectable by Sanger in saliva. Therefore, even if only supposed germline mutation was targeted by Sanger, absence of acquired mutation could not be verified and tumor infiltration in saliva cannot be definitively excluded. Two cases are nevertheless interesting:

- Patient 5: Bone marrow at diagnosis showed two mutations of *DDX41*: G173R (VAF=53%) and a R525H (VAF=20%), associated with a *TET2* mutation (VAF=98%). Bone marrow collected for follow-up after allogenic HSC transplantation with a relative showed acquisition of a SNP from the donor (homozygous *SETBP1*: Y1303S) and presence of *DDX41* G173R mutation. This patient next presented a relapse of his disease after transplantation, with *DDX41* G173R and R525H mutation, and presence of *SETBP1* homozygous Y1303S. Sanger sequencing was performed on saliva collected two months later, which showed presence of *DDX41* G173R. Thus, it was not possible to affirm that saliva was not contaminated by tumoral cells (*DDX41* R525H not targeted), and we could not conclude if mutation detected in saliva was carried by patient or brought by the transplantation with a carrier relative (*SETBP1* SNP not targeted by Sanger).
- Patient 18: Bone marrow at diagnosis show presence of *RUNX1* E223* (VAF=45%; depth=5925X) and *SRSF2* P95H (VAF=46%, depth=483X) mutations. NGS performed on saliva collected two months later (and without any treatment) detected only *RUNX1* E223* mutation (VAF=42%, depth=361X). Due to low sequencing depth on saliva, it is probable that even if *SRSF2* P95H mutation was present at heterozygous state, bioinformatic pipeline would have filtrated this mutation (probable depth <50X). Thus, saliva was considered as potentially infiltrated, so “inconclusive”. Unfortunately, patient died of endocarditis before we could collect hair follicles or skin fibroblast, so germline nature of *RUNX1* E223* mutation could not be verified.

## 4 Discussion

Predisposition to myeloid disorders is a recent field of genetics, especially since the definition of a specific category of tumors by WHO in 2016 (19). This definition of “Myeloid Neoplasms with Germline Predisposition” category (2, 3) was based on several studies, perfectly summarized in the review of Klco and Mulligan in 2021 (20).

Due to absence of strong recommendations, strategy for diagnosis of predisposition to myeloid malignancies was highly variable between our patients. Some of them benefited of Sanger sequencing on saliva, other ones of HTS. That was one of the weaknesses of our study. For some cases, only saliva samples collected at remission were informative (24% of our cohort), and retrospectively, obtaining more samples at remission would probably reduce the rate of inconclusive saliva samples.

Saliva was preferably studied because its collection is less invasive than skin biopsy. However, we showed that in case of allogenic HSC transplantations, analysis on DNA from skin fibroblasts are required. These results are concordant with methodology used by other authors, such as Churpek et al. (21) and Li et al. (22) who used either skin biopsy, T-lymphocytes from blood, or buccal swab to affirm germline nature of the detected variants. Our initial workflow thus retrospectively appeared unappropriated to patients that are being evaluated for HM predisposition. It was centered on Sanger sequencing of saliva samples, similarly to patients with a suspected hereditary predisposition to solid tumors.

Most saliva samples collected from affected patients (48%) showed obvious signs of infiltration by tumoral cells. In these cases, mutational profiles (mutations and VAF) were comparable between medullary and salivary compartments. Moreover, analysis performed on blood (patient ID4 and 25) even showed an infiltration higher in saliva than in blood; and case of patient ID13 interestingly showed different mutations in saliva compared to bone marrow at diagnosis, which could be compatible with sub-clonal mutations not detectable in medullary compartment.

In order to resolve the problem of infiltration in saliva, we could test extraction of DNA from hair follicle in the 4 most recently included patients (ID1, 10, 12 and 14). Results were encouraging, as they confirmed the results obtained on the skin for patient ID12 and avoided skin biopsy for the others (ID1, 10 and 14). These results need to be confirmed on larger cohort of patients and could even be used *a posteriori* to conclude on the saliva samples that we considered as “unconclusive” (28% of our patients).

Concerning the relatives, we showed that saliva was usable, after verification of absence of hemopathy signs by standard blood count and clinical examination. In some cases, detection of mutation in relatives proved the germline nature of mutation detected at diagnosis in index case. For example, Sanger sequencing performed on patient ID6’s son (ID37) to determine his eligibility as a donor for HSC allograft, showed that he carried the heterozygous *DDX41* L283Cfs*21 mutation. Consequently, his status was known at 32 years old, while HTS performed in bone marrow at diagnosis and in his father’ saliva showed identical results, suggesting a significant infiltration of saliva by tumoral cells. Thus, segregation of mutation in this family proved germline nature of *DDX41* variant detected in patient ID6. In the context of intrafamilial allogenic HSC transplantations, early testing of relatives appears to be ethically acceptable but is more debatable out of those situations, considering the penetrance of *DDX41* traits, which is negligible until 40 years of age (6).

**Figure 2:**
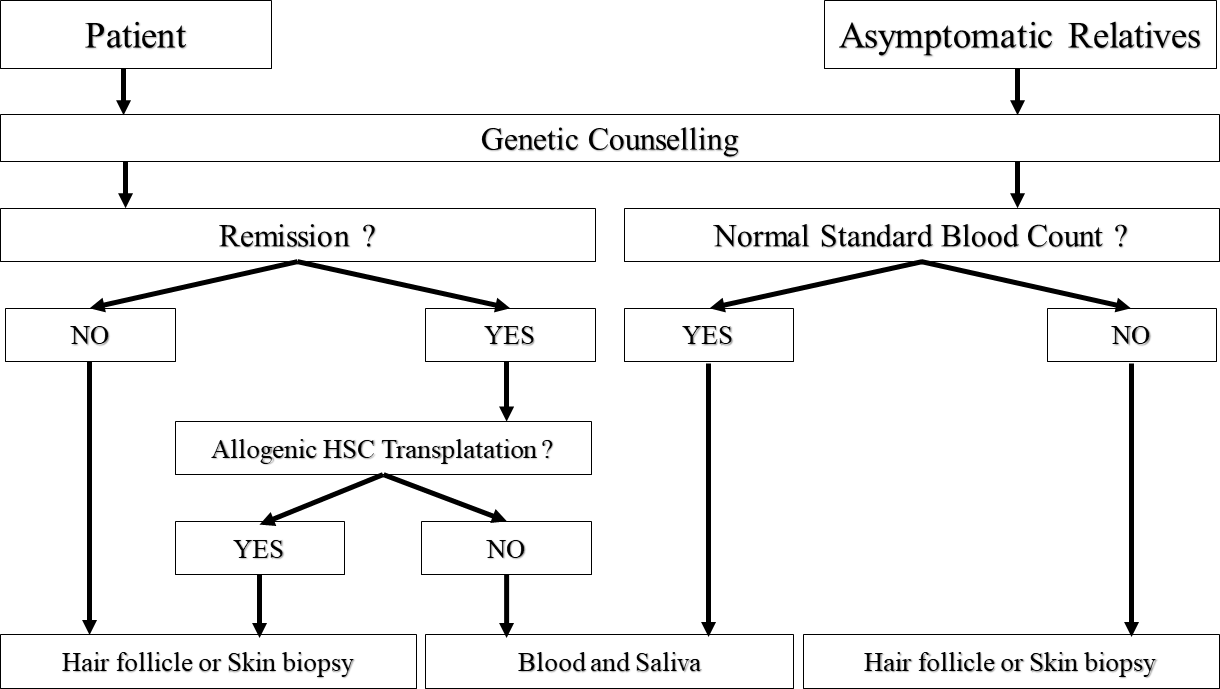
Algorithm proposed for diagnostic. HSC = Hematopoietic Stem Cells. Relatives with abnormal standard blood count should be considered as patients. Conversely, patients at remission without HSC transplantation can be analyzed as asymptomatic relatives.

In our Institution, fibroblasts are not cultured by hematology biologists, but in the Genetics unit. Availability of skin biopsies for patients followed up by hematologists is thus conditioned by another unit, with its own cell culture activity. Consequently, and despite the small cohort of patient available, our local experience led us to define an optimized algorithm for the diagnosis of hereditary predisposition to HM, to limit the use of skin biopsy to patients whom it is indispensable. (**Figure 2**).

This algorithm, easily usable in clinical routine, focuses on the importance of collecting patients’ samples at remission if possible. Due to difficulties of interpretations encountered in patients treated with allograft, the use of saliva should be limited to relatives (Sanger sequencing) or patients at remission (HTS) without antecedent of transplantation, even if the donor was unrelated to patient. In case where saliva is not usable, or when patients’ samples cannot be collected at remission, we suggest the use of hair follicles that are less invasive to collect, but biopsy remains the gold standard until a larger cohort confirms our results.

## 5 Conclusion

Our study showed that HTS is an efficient tool for detecting tumoral cells in patients’ saliva samples or assessing donor contribution in case of HSC transplantation. In addition, we showed that saliva can be used in the field of predisposition to malignant myeloid hemopathies, but cautions are needed to ensure proper interpretation of the results.

## Data availability statement

The original contributions presented in the study are included in the article/**Supplementary Material**. Further inquiries can be directed to the corresponding author.

## Ethics statement

The studies involving human participants were reviewed and approved by Comité d’éthique du CHU de Limoges. The patients/participants provided their written informed consent to participate in this study.

## Author contributions

AP: Planned the experiment, analyzed the results, and wrote the manuscript. SB: Technical and biological validation of the results, and critical revision of the manuscript. DR: Technical and biological validation of the results, and proofread the manuscript. JC: Technical and biological validation of the results, and proofread the manuscript. BD: Patients and relatives genetic counselling, and proofread the manuscript. PT: Patients clinical diagnostic and follow-up. SG: patients clinical diagnostic and follow-up. LV: Patients and relatives genetic counselling. MR: patients and relatives genetic counselling. JF: Proofread the manuscript. CY: Critical revision of the manuscript. NG: Technical and biological validation of the results, and critical revision of the manuscript. All authors contributed to the article and approved the submitted version.
