# Supplementary material for: Hereditary predisposition to malignant myeloid hemopathies: Caution in use of saliva and guideline based on our experience
Source: Front Oncol. 2023 Feb 27;13:1120829. doi: 10.3389/fonc.2023.1120829 (PMC10008954; doi:10.3389/fonc.2023.1120829)
Supplement: Supplementary file 1 [file DataSheet_1.zip › Data Sheet 1/Supplementary Figure.docx]

# Supplementary Figure

**
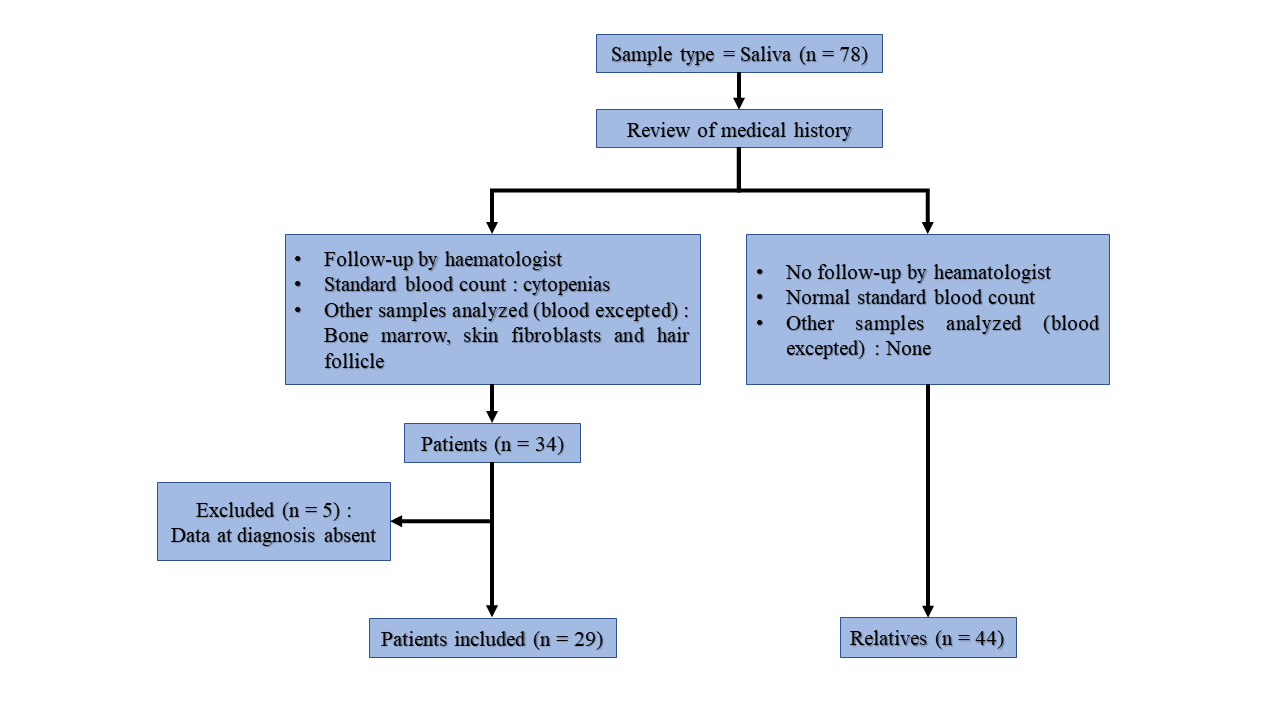
**

**Figure S1: Flowchart of our study.** Inclusion of patients and relatives was retrospective. Patients were distinguished from their relatives by study of medical history and review of analysis available.
